# Of the Treatmet of Croup by the Inhalation of Lime-Water

**Published:** 1866-01

**Authors:** 


### OF THE TREATMET OF CROUP BY THE INHALATION OF LIME-WATER.

M. Kuchenmeister, of Dresden, has stated that diphtheritic membranes are rapidly dissolved in lime-water; and this statement has been confirmed by M. Biermer, the Professor of Clinical Medicine in the University of Berne, who has repeated the experiment before the students of his class.

The *Brit. For. Med. Chir. Review*, says that some pseudo-membraneous exudations, of considerable extent and thickness, were placed in a small glass of lime-water, and in the space of from ten to fifteen minutes, and before the eyes of the students, they disappeared, leaving only a very slight sediment at the bottom of the glass. M. Biermer was therefore induced to apply the lime-water locally in a living patient, and he has published the results, which were quite satisfactory. The patient was a girl, aged seventeen, admitted into the hospital of Berne for croup, which has lasted four days. When she was admitted, she was nearly choked, cyanotic, and insensible, and she threw up portions of membrane only by means of the administration of some very strong irritant medicines. The symptoms of laryngeal constriction still continued, together with distressing dyspnœa; and pulverized water was employed to moisten the respiratory passages. The water employed, which was at first hot, and then boiling, produced considerable amelioration; and M. Biermer, having previously tried the experiment mentioned above, with the false membrane and lime-water, supplied the pulverizer with lime-water. The improvement was evident as soon as the inhalations were commenced; the expectoration changed its character, and became purulent; the cough gradually disappeared, and the fever abated; and only hoarseness and a slight cough remained during the convalescence, which terminated in a complete cure. M. Biermer, and all those who watched the progress of the case, were convinced that the inhalations had a solvent effect upon the false membranes; but the professor does not recommend an exclusive adoption of this local treatment, which softens and detaches the exudations, but does not reach the cause of the disease, which must be combatted by constitutional remedies, calomel being considered the chief. The plan of M. Biermer has been followed by other practitioners; and M. Kuchenmeister has published a case of diphtheritic pharyngo-laryngitis in a child of three years and a-half old, treated in the same manner with complete success. Dr. Brauser, of Ratisbon, has also lately published a case of croup in a child of four years and a-half old, treated in the same manner, and perfectly cured. M. Biermer insists particularly on the necessity of using the injections hot.—*Buffalo Med. & Surg. Journal.*

